# Potential predictive effect of mechanical properties of the plantar skin and superficial soft tissue, and vibration perception on plantar loading during gait in individuals with diabetes

**DOI:** 10.1186/s12891-023-06851-7

**Published:** 2023-09-06

**Authors:** Renan L. Monteiro, Tina J. Drechsel, Jane Suelen S. P. Ferreira, Claudio Zippenfennig, Isabel C. N. Sacco

**Affiliations:** 1 Department of Physical Therapy, Speech, and Occupational Therapy, School of Medicine, University of São Paulo, São Paulo, Brazil; 2 Department of Health and Biological Science, Federal University of Amapá, Macapá, Brazil; 3 Department of Human Locomotion, Institute of Human Movement Science and Health, Chemnitz University of Technology, Chemnitz, Germany; 4 Departamento de Fisioterapia, Fonoaudiologia e Terapia Ocupacional da Faculdade de Medicina da Universidade de São Paulo, Rua Cipotânea, 51 - Cidade Universitária, São Paulo, 05360-160 Brazil

**Keywords:** Diabetic neuropathies, Plantar pressure, Gait, Vibration perception threshold, Sensory perception, Skin hardness, Skin stiffness, Skin thickness

## Abstract

**Background:**

This exploratory study aimed to investigate the extent to which mechanical properties of the plantar skin and superficial soft tissue (hardness, stiffness, and thickness) and vibration perception thresholds (VPTs) predict plantar pressure loading during gait in people with diabetes compared to healthy controls.

**Methods:**

Mechanical properties, VPTs, and plantar loadings during gait at the heel and first metatarsal head (MTH) of 20 subjects with diabetes, 13 with DPN, and 33 healthy controls were acquired. Multiple regression analyses were used to predict plantar pressure peaks and pressure-time integrals at both locations based on the mechanical properties of the skin and superficial soft tissues and VPTs.

**Results:**

In the diabetes group at the MTH, skin hardness associated with 30-Hz (R^2^ = 0.343) and 200-Hz (R^2^ = 0.314) VPTs predicted peak pressure at the forefoot. In the controls at the heel, peak pressure was predicted by the skin thickness, hardness, and stiffness associated with 30-Hz (R^2^ = 0.269, 0.268, and 0.267, respectively) and 200-Hz (R^2^ = 0.214, 0.247, and 0.265, respectively) VPTs.

**Conclusion:**

The forefoot loading of people with diabetes can be predicted by the hardness of the skin when combined with loss of vibration perception at low (30-Hz) and high (200-Hz) frequencies. Further data from larger sample sizes are needed to confirm the current findings.

**Supplementary Information:**

The online version contains supplementary material available at 10.1186/s12891-023-06851-7.

## Background

Currently, there are estimated 537 million people with diabetes worldwide, 50% of whom have not yet been diagnosed [1]. In addition, it is estimated that between 12% and 50% of people with diabetes mellitus have some degree of diabetic peripheral neuropathy (DPN) [2, 3], which damages the peripheral and autonomic nervous systems [4], and results in sensorimotor dysfunctions, such as: loss of protective sensation [5], increased vibration perception threshold (VPT) [6], changes in mechanical skin properties [7, 8], increased joint stiffness [9], reduction of foot-ankle joint mobility [10], atrophy of the intrinsic and extrinsic foot-ankle muscles [11–14] and muscles of the lower limbs [15–17].

All these sensorimotor dysfunctions (e.g., decreases in vibration perception and changes in mechanical skin properties) compromise the proper foot-ankle joint mobility during locomotor activities and the proper foot rollover mechanism, resulting in increased plantar loadings [18–20]. It is already known that in the later stages of diabetes progression, there is a greater sensorimotor dysfunction, greater changes in the foot rollover, and significant changes in plantar pressure distribution [20, 21], such as high peak pressure during gait, which is a well-known predictor of plantar ulcers, together with other factors such as foot deformity, loss of sensitivity, and peripheral arterial disease [22–24].

Considering the scenario described, the main hypothesis of this study is that mechanical properties of the plantar skin and superficial soft tissue can predict plantar loading, as suggested by Allan, 2022 [25]. In addition, we hypothesized that there is a relationship between plantar loading exposure during foot rollover and stiffness/hardness of the plantar superficial soft tissues due to keratinization [26, 27]. Keratinization is the process of cell differentiation, in which the keratinocytes differentiate structurally and functionally in response to increased shear stresses under the plantar surface, which in turn causes the formation of calluses, an adaptive, thickening reaction of the keratinized layer of the epidermis [27]. Based on this main hypothesis, we designed an exploratory study to examine the relationships among biomechanical- and diabetes-related factors, as well as mechanical skin properties, in order to obtain foundations to better address the question posed in this work.

There is some evidence that supports these explanations about the relationship between mechanical properties and changes in plantar loading during foot rollover of people with diabetes. Klaesner et al. [28] found an increase in the plantar superficial soft tissue hardness in people with diabetes, DPN, and a history of foot ulcers, concluding that skin hardness might be an important risk factor for plantar pressure ulcers in this population. Corroborating these findings, Piaggesi et al. [29] showed a higher degree of skin hardness in people with DPN before any ulceration was present compared to people with diabetes without DPN and healthy controls. Zippenfennig et al. [8] observed harder plantar skin at the heel in individuals with diabetes and DPN compared to healthy controls. Chao et al. [7] also observed greater thickness and stiffness of plantar superficial soft tissue in people with diabetes and a higher proportion of harder skin in people with DPN. Based on the data presented, it is clear that even before DPN has manifested, the mechanical properties of the plantar skin and superficial soft tissue are already changed in people with diabetes, which demonstrates the importance of assessing plantar mechanical properties for earlier detection of diabetes changes in foot regions commonly affected by higher loads and ulcer prevalence.

Increased superficial soft tissue stiffness, skin thickness and hardness, such as in a callus, can be considered a self-protection mechanism of the foot. In healthy people, a callus does not compromise the ability to perceive vibrotactile stimuli [30]. From a pathological perspective, changes in the skin’s mechanical properties in people with diabetes and DPN are caused by the disease itself. In contrast to healthy people, these changes may be a compensatory mechanism in response to the loss of sensitivity up to a certain progression of the disease: the harder the skin in people with DPN, the better the perception of 30-Hz vibrations, and the thicker the skin in people with diabetes without DPN, the better the perception of 200-Hz vibrations [8]. Increased spatial summation of Pacinian corpuscles could provide an explanation for the improvement in vibration perception with increasing in the skin thickness in people with diabetes [8]. As a compensatory mechanism, increasing skin hardening in people with diabetes and DPN could lead to a better and wider spread of vibrations, stimulating a higher quantity of remaining Meissner corpuscles [8].

Considering that the mechanical properties of plantar tissues are changed due to diabetes even before DPN has manifested, this would definitely have an effect in the plantar loading parameters during gait. We assume as a premise in our study that the plantar pressure distribution would be changed by an alteration in the sensorial perception and foot rollover during gait, which in turn might be a result of changes in the mechanical properties of the foot tissues, such as a reduction in the viscoelasticity of conjunctive tissues around foot joints, foot-ankle muscle atrophy, and foot deformities. Therefore, the main aim of this study was to investigate the predictive effect of the mechanical properties of the plantar skin and superficial soft tissue (hardness, stiffness, and thickness) and the VPT at 30-Hz and 200-Hz on plantar pressure loading during foot rollover in gait in individuals with diabetes compared to healthy controls.

## Methods

### Design

This is an exploratory study designed to investigate the relationship between VPTs and mechanical skin properties in people with and without diabetes [8] and to determine to what extent low (30-Hz) and high (200-Hz) VPTs could be used for the early detection of DPN [6]. A detailed description of that study was published elsewhere [6]. The study was approved by the ethical committee of the School of Medicine of the University of Sao Paulo (Protocol 1.464.870). All subjects were informed in detail about the nature of the study and gave their written informed consent prior to participation. All methods were carried out in accordance with declaration of Helsinki.

### Participants

People with diabetes were recruited from a primary care center in the city of Sao Paulo, *Ambulatório Médico de Especialidades Maria Zélia.* Data were collected on 20 subjects with type 1 and 2 diabetes (diabetes group) and 33 healthy control participants (Table 1). Participants were classified as DPN based on a fuzzy decision support system [17]. The non-inclusion criteria were: partial or total amputations of the foot (with the exception of a single toe that was not the hallux); diagnosed neurological impairments due to stroke, cerebral palsy, or poliomyelitis; dementia or inability to give consistent information; major vascular complications (venous or arterial ulcers); severe retinopathy; severe nephropathy causing edema or requiring hemodialysis; presence of plantar ulcers at the time of evaluation; and inability to walk independently without pain or the use of an assistive device.

**Table 1:** Demographic and anthropometric characteristics of the studied groups

Group	Gender (F/M)	Age (yrs old) (F / M)	Height (m) (F / M)	Body mass (kg) (F / M)	Diabetes duration (years)
**Diabetes group** **(n = 20)**	13 / 7	56.7 ± 12.1 / 47.0 ± 19.0	1.6 ± 0.1 / 1.7 ± 0.1	77.8 ± 13.9 / 78.0 ± 13.3	14.03 ± 10.09 / 7.2 ± 7.9
**Control group** **(n = 33)**	20 / 13	56.2 ± 14.6 / 54.9 ± 17.2	1.6 ± 0.1 / 1.8 ± 0.1	65.1 ± 11.2 / 77.7 ± 8.8	-

The subjects were assessed for the following parameters. (1) Plantar mechanical properties were shore hardness values of the plantar skin measured using a Shore 00 Durometer (AD-100; Checkline Europe BV, Enschede, Netherlands), superficial soft tissue stiffness measured using a custom-built indentometer (Chemnitz University of Technology, Chemnitz, Germany), and epidermal thickness measured using a handheld ultrasound device (Model L7; Clarius Mobile Health Corp., Burnaby, Canada). (2) VPT of two measurement frequencies (30- and 200-Hz) were collected using a modified vibration exciter (Mini-Shaker type 4180; Brüel & Kjaer Vibro GmbH, Darmstadt, Germany). (3) Pressure distribution during gait was measured with a pressure measuring plate (emed-q100; novel, Munich, Germany). All measurements were performed at two plantar areas (heel and first metatarsal head [MTH]) of one foot, which was randomly assigned. For each measurement, the mean out of three measurement trials per plantar area was used for statistical purposes.

### Mechanical properties of the plantar skin and superficial soft tissue

All mechanical properties of the plantar skin and superficial soft tissue were measured in a prone position. The plantar skin hardness was determined by a Shore 00 Durometer, which is a simple and cost-effective measurement device capable of determining a material’s resistance to indentation. Hardness was defined as the indentation depth created by a defined pressure [30], and a greater Shore value indicated higher resistance to indentation (the Shore scale ranges from 0 [softest] to 100 [hardest]) [31, 32]. The analogue scale of the device provided arbitrary units based on the penetration depth of the device probe (diameter of 2.4 mm) applied perpendicularly.

The mechanical stiffness of the plantar superficial soft tissue was determined using a custom-built indentometer. Its circular probe (diameter of 11.3 mm) was pressed against the plantar skin until a force of 30 N was reached. Then, the device tracked the indentation depth of the probe and exerted force. The plantar tissue stiffness was defined by the slope of the relationship between indentation depth and force [30].

**Fig. 1:**
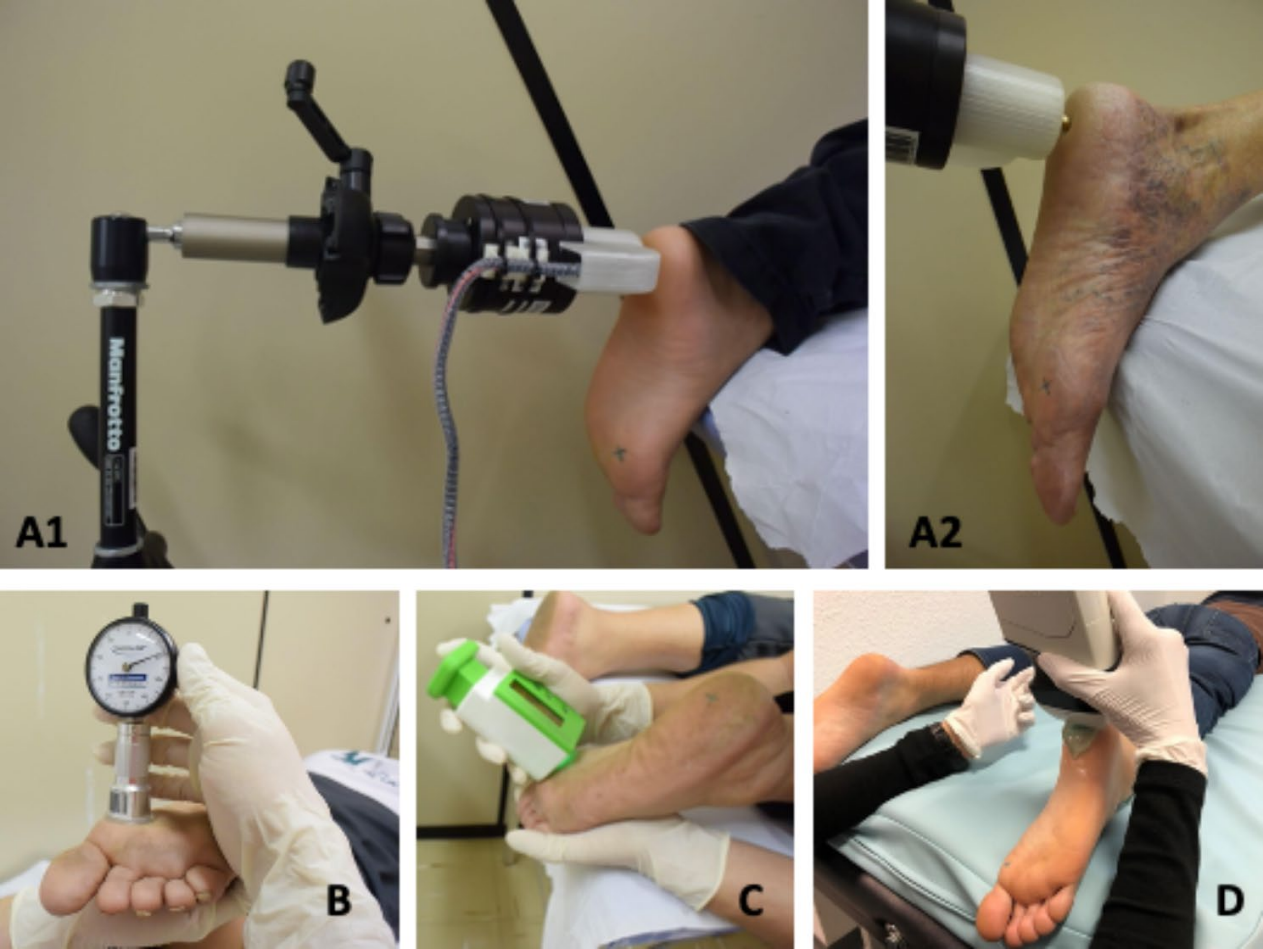
Measurement of the mechanical properties of plantar skin and superficial soft tissue. **A1** and **A2**: Modified vibration exciter (Mini-Shaker); **B**: Custom-built indentometer; **C**: Shore 00 Durometer; **D**: Handheld ultrasound device

The plantar epidermal thickness was determined by a handheld ultrasound device (Model L7; Clarius Mobile Health Corp., Burnaby, Canada). The scanner’s transmission frequency was optimized at 10 MHz with automatic adjustment according to depth. The sharpest out of three ultrasound images were evaluated by two independent investigators using ImageJ planimeter software (NIH, Maryland, USA) [33]. The distance (in mm) was measured between the two superficial hyperechoic lines representing the borders of the epidermis [34] (Fig. 1).

### Vibration perception threshold

VPTs were also measured in a prone position at two different frequencies (30- and 200-Hz) on the same foot and plantar locations as the previous measurements of the mechanical properties of the plantar skin and superficial soft tissue. These two frequencies are considered ideal for measuring Meissner (Rapidly Adapting - RAI) and Pacinian (RAII) corpuscles [35–37]. Both frequencies and anatomical locations tested for each subject had their order of testing randomized. Using a swivel arm, the probe (diameter of 7.8 mm) of a modified vibration exciter (Mini-Shaker type 4180) was placed perpendicularly at the foot [6, 8]. The vibration exciter was powered by a power bank (XTPower MP-3200; Batteries and Power Solutions GmbH, Ellwangen, Germany). The vertical movement of the vibration exciter’s probe was calibrated before the measurements using a high-precision capacitive position sensor (CS05; Mirco-Epsilon Messtechnik GmbH & Co. KG, Ortenburg, Germany). The vibration amplitude (in µm) was calculated using an acceleration sensor (MMA2240KEG; NXP Semiconductors Netherlands B.V., Eindhoven, Netherlands) [6, 8]. The contact force of the probe was precisely controlled (intended range: 1.0 ± 0.2 N) by means of an integrated force sensor (DS050A9; disynet GmbH, Brüggen-Bracht, Germany). A self-written LabVIEW program ran a customized VPT protocol inspired by Mildren et al. [32]The VPT was determined from the means of the last recognized and last unperceived vibration stimuli [6, 8].

### Plantar pressure distribution during gait

**Fig. 2:**
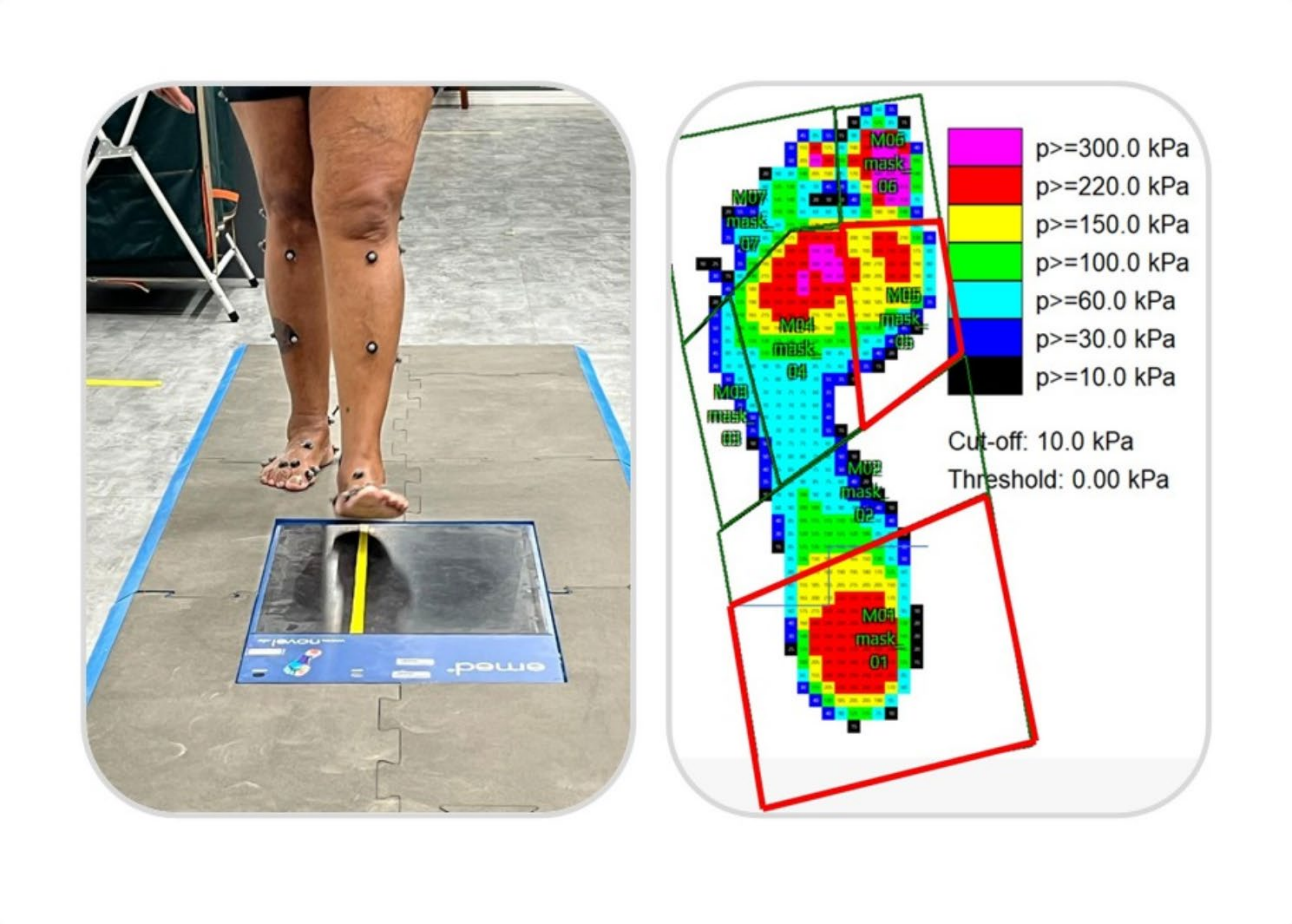
Measurement of plantar pressure distribution during gait and the foot masks used. The areas in red were the areas analyzed

The emed-q100 pressure platform (emed-q100; novel, Munich, Germany, 4 sensors/cm^2^) was used to assess plantar loading variables during gait. After familiarization with the laboratory and platform, participants walked barefoot across the platform at a similar self-selected speed among groups (Diabetes Group (n = 20): 1.0 ± 0.2 m/s, DPN Group (n = 13): 0.9 ± 0.2 m/s, Control Group (n = 33): 1.1 ± 0.2 m/s, p ANOVA = 0.091) for a distance of 6 m. The mean of three trials was used for statistical purposes. The measurements of the same foot that was assessed for the mechanical properties of the plantar skin and superficial soft tissue and VPTs were used for the plantar pressure analysis. We analyzed peak pressure and pressure-time integral in both plantar areas to represent plantar loadings using a geometrical footprint mask from 2 areas: heel and first metatarsal head (Fig. 2) (multimask software; novel, Munich, Germany).

### Statistical analysis

VPTs are recorded on a ratio scale, which may lead to a heteroscedastic and non-normal distribution [38]. To correct this distribution, VPT data were transformed using the natural logarithm [38]. Based on the normal distribution of the data, multiple regression analyses using the hierarchical method were conducted to determine the prediction of plantar pressure loading parameters during gait in both anatomical locations based on the mechanical properties of the plantar skin and superficial soft tissue and VPTs. The four dependent variables (peak pressure at heel and MTH; pressure-time integrals at heel and MTH) that were significantly correlated with one of the ten independent variables — hardness at (1) heel and (2) MTH, stiffness at (3) heel and (4) MTH, thickness at (5) heel and (6) MTH, 30-Hz VPT at (7) heel and (8) MTH, and 200-Hz VPT at (9) heel and (10) MTH — were then inputted into a multivariable linear regression hierarchical model (Pin = 0.05, Pout = 0.10) to determine the amount of variance in the pressure parameters that was explained by the correlated independent variables. Each model was limited to only one dependent and two independent variables, as our sample size did not allow for the inclusion of more independent variables [39]. In addition, the models were built for each group separately as we intended to understand the role of the dependent variables in people with and without diabetes. The prediction analysis was performed only with the diabetes group and healthy controls.

The variables contained in the final model were used to generate Eq. 1 following the multiple regression model:

1 $$Y = a + b1.X1 + b2.X2$$

where *Y* is the dependent variable to be predicted, *a* is the constant of the regression, and *b* corresponds to the unstandardized regression coefficients of each independent variable (*X*).

Effects due to multicollinearity were limited by ensuring that Pearson’s coefficients between the input variables in the regression model were less than 0.8 [40]. The Durbin-Watson test was used to assess the presence of autocorrelation prior to the modelling procedure. Variance inflation factors were used to assess multicollinearity, and the suitability of the regression model was evaluated by examining the plots of the residuals against the predicted values. All data analyses were performed using SPSS (version 28.0.10; IBM Corp, Armonk, NY) with a significance threshold of alpha = 5%.

## Results

For the diabetes group, the peak pressure at the forefoot was predicted by the skin hardness associated with 30-Hz VPT, F(2,17) = 4.428, p = 0.028, R^2^ = 0.343, and 200-Hz VPT, F(2,17) = 3.894, p = 0.041, R^2^ = 0.314, at MTH. Both skin hardness, F(2,17) = 4.350, p = 0.030, R^2^ = 0.339, and superficial soft tissue stiffness, F(2,17) = 4.309, p = 0.031, R^2^ = 0.336, associated with 30-Hz VPT, predicted peak pressures at the heel. In addition, the association of 30-Hz VPT with superficial soft tissue stiffness, F(2,17) = 4.626, p = 0.025, R^2^ = 0.352, predicted the pressure-time integral at the heel (Table 2).

Unlike the diabetes participants, for the controls, the soft plantar tissue thickness at the heel associated with 30-Hz VPT, F(2,28) = 5.278, p = 0.011, R^2^ = 0.274, and 200-Hz VPT, F(2,28) = 3.838, p = 0.034, R^2^ = 0.215, predicted the peak pressure during gait. The peak pressure at the heel was also predicted by the combination of the skin hardness with 30-Hz VPT, F(2,30) = 5.521, p = 0.009, R^2^ = 0.269, and 200-Hz VPT, F(2,30) = 4.937, p = 0.014, R^2^ = 0.248. In addition, peak pressure at the heel was predicted by the combination of the superficial soft tissue stiffness with the 30-Hz VPT, F(2,30) = 5.508, p = 0.009, R^2^ = 0.269, and 200-Hz VPT, F(2,30) = 5.448, p = 0.010; R^2^ = 0.266 (Table 2). In the controls, none of the independent variables were shown to have significant predictive value at the MTH.

**Table 2:** Multiple regression models predicting plantar pressure parameters from mechanical properties of the plantar skin and superficial soft tissue in the diabetes and control groups

Y	a	b1	X1	b2	X2	p value / R^2^	R^2^change
**DIABETES GROUP**							
Peak pressure at MTH	114.4	9.2	hardness at MTH	-20.6	30-Hz VPT	0.028 / 0.343	0.029
Peak pressure at MTH	75.3	8.7	hardness at MTH	-1.4	200-Hz VPT	0.041 / 0.314	0.000
Peak pressure at heel	64.5	3.6	hardness at heel	28.1	30-Hz VPT	0.030 / 0.339	0.156
Peak pressure at heel	32.3	25.2	stiffness at heel	69.2	30-Hz VPT	0.031 / 0.336	0.170
Pressure-time integral at heel	-3.3	7.9	stiffness at heel	28.1	30-Hz VPT	0.025 / 0.352	0.228
**CONTROL GROUP**							
Peak pressure at heel	285.4	-0.9	hardness at heel	133.1	30-Hz VPT	0.009 / 0.269	0.266
Peak pressure at heel	513.4	2.1	hardness at heel	81.7	200-Hz VPT	0.014 / 0.248	0.245
Peak pressure at heel	241.1	3.9	stiffness at heel	131.7	30-Hz VPT	0.009 / 0.269	0.231
Peak pressure at heel	406.7	30.6	stiffness at heel	77.2	200-Hz VPT	0.010 / 0.266	0.229
Peak pressure at heel	472.7	-245.1	thickness at heel	123.1	30-Hz VPT	0.011 / 0.274	0.245
Peak pressure at heel	660.7	-105.5	thickness at heel	69.3	200-Hz VPT	0.034 / 0.215	0.186

*Y* is the dependent variable to be predicted, *a* is the constant of the regression, and *b* corresponds to the unstandardized regression coefficients of each independent variable *X*. Abbreviations: MTH – First Metatarsal Head. VPT – Vibration Perception Threshold

Differences between healthy participants and participants with diabetes in terms of the plantar mechanical properties, VPTs, peak pressures, and pressure-time integrals are provided in the supplementary file [see supplementary file 1].

## Discussion

This study investigated the predictive value of biomechanical plantar tissue properties and VPTs with respect to plantar pressure loads occurring during foot rollover while walking in people with diabetes. The main results showed that for individuals with diabetes skin hardness in combination with VPTs of both measured frequencies at the MTH predicted peak pressures in the forefoot. Additionally, the combination of 30-Hz VPT with hardness and stiffness predicted heel pressure loadings. As for the controls, the only plantar region for which plantar loading parameters could be predicted was the heel using a combination of the 30- and 200-Hz VPTs with the skin hardness, stiffness, and thickness.

In people with diabetes, 31% and 34% of the peak pressure at the MTH was explained by skin hardness combined with VPTs (30 and 200-Hz, respectively). The prediction analysis included correlation analysis, revealing a moderately positive correlation between plantar pressure and hardness variables. This finding aligns with the results of a previous study conducted by Allan et al. [25]. This result was not found in the healthy controls. Thus, alterations resulting from diabetes, such as changes in the mechanical properties of the skin and superficial soft tissue [7, 8, 28, 29] and increased VPTs at the forefoot area [6, 8, 41, 42]are both responsible for the changes in plantar loading exposure at the forefoot, which is an anatomical area that is highly affected by plantar pressure ulcers [43–45].

Hardness, stiffness and thickness of the skin, which are related to callus formation, can universally predict the peak pressure and pressure-time integral at the heel. Regardless of diabetes or DPN, all individuals usually experience increased pressure on the sole of their foot due to walking’s mechanical demands. It is probable that, as a result of the keratinization process induced by the increased vertical and shear stresses under the plantar surface common at the heel, there was an adaptive, thickening reaction of the plantar skin under this area of all groups, and the relationship could be observed in our predictive models for controls and people with diabetes [46].

However, at the forefoot, where the foot rolls forward when walking and the loads are smaller than at the heel, diabetes may play a major role in determining the relationship between peak pressure and pressure-time integral with skin mechanical properties. The following is important to note: in our sample, the 200-Hz VPT at both anatomical locations (heel and MTH) and the 30-Hz VPT at the MTH of people with diabetes were lower than those of healthy control subjects [6]. In addition to influencing the keratinization process, and thus the hardness and stiffness of the skin [8], these sensory changes could also have influenced the rolling of the foot. This might have changed the pressure patterns during walking [19, 20, 45], explaining the relationships found in the predictive models.

We observed previously a significant correlation between VPT and DPN severity determined by the Fuzzy score [6], where the more severe DPN, the greater the skin properties changes [7, 8] and, consequently, the plantar pressure distribution during gait. This finding could explain the increase of the predictive value of the equation, after VPT was added.

Although we cannot determine causal relationships between changes in the mechanical properties of the plantar tissues and changes in the plantar pressure distribution in people with diabetes, there are some hypotheses that could justify such a prediction of plantar loading through the mechanical properties of the skin, particularly the hardness of the skin. There is evidence that people with diabetes without DPN have alterations in the neuromuscular component such as (1) change in motor control variability and muscle force complexity [18]; (2) change in muscle fiber conduction velocity [47, 48]. In addition, loss of neuromuscular components’ properties and function as a progressive alteration resulting from diabetes advancement leads to foot and toe deformities and muscle atrophy, which in turn results in persistent abnormal pressure under the foot [49]. With this in mind, it is clear that motor and locomotor alterations due to muscle dysfunction can be observed in people with diabetes without DPN. Changing the foot rollover during gait due to these musculoskeletal alterations may lead to changes in plantar loading during gait. The cells of skin react to the persistent higher plantar loading exposure by increasing keratinization and turning into a callus. As a result, increased skin hardness and stiffness in people with diabetes can explain the findings of the prediction models in the diabetes group.

The present study is not without limitations. This was an exploratory study, which did not allow us to infer a causal relationship. Further studies could advance in this context if a longitudinal design is adopted. Although our results showed interesting relationships between two or three variables, we should emphasize that the study has a small sample size, and such a limited number of participants per group limited the inclusion of more independent variables in the prediction models. Further studies should concentrate the prediction analysis with participants with DPN to investigate the effect of the progressive sensorial losses in the prediction of plantar pressure variables from mechanical properties of the plantar superficial soft tissue. Another limitation was that our participants’ history of previous podiatric treatment and corresponding callus removal was not investigated before our measurements. Faced with such limitations, interpretations of the regression results may be compromised. Thus, further data are needed to confirm the hypothesis raised in our study.

## Conclusion

In summary, we showed that the forefoot loadings in people with diabetes can be predicted by the hardness and stiffness of the skin, but only when combined with loss of vibration perception. Plantar loadings at the heel can be predicted by a combination of VPT and plantar mechanical properties for both healthy individuals and those with diabetes. Thus, diabetes may play a role in determining pressure patterns during gait at the forefoot but not at the heel. Possibly, the measurement of both mechanical skin properties and vibration sensitivity could become valuable additional tools to forecast diabetic foot ulcerations [50]. However, these findings need to be confirmed in studies with larger sample sizes so that we can reinforce or refute the current findings.

### Electronic supplementary material

Below is the link to the electronic supplementary material.

Supplementary Material 1

## Data Availability

The datasets used and/or analyzed during the current study available from the corresponding author on reasonable request.

## Abbreviations

DPN: Diabetic Peripheral Neuropathy
VPT: Vibration Perception Threshold
MTH: First Metatarsal Head
RAI: Rapidly Adapting (Meissner corpuscles)
RAII: Rapidly Adapting (Pacinian corpuscles)
