# Supplementary material for: Potential predictive effect of mechanical properties of the plantar skin and superficial soft tissue, and vibration perception on plantar loading during gait in individuals with diabetes
Source: BMC Musculoskelet Disord. 2023 Sep 6;24:712. doi: 10.1186/s12891-023-06851-7 (PMC10483699; doi:10.1186/s12891-023-06851-7)
Supplement: Supplementary file 1 — Supplementary Material 1 [file 12891_2023_6851_MOESM1_ESM.docx]

**Supplementary file 1**

**Title of data:** Differences between diabetic and healthy participants regarding the plantar mechanical properties, VPT, peak pressure, and pressure-time integral.

**Description of data:** The hardness (p=0.038) and thickness (p=0.011) at the heel were greater and the peak pressure (p<0.001) was smaller for diabetes participants compared to controls (Supplementary Table). At the MTH, peak pressure was higher in diabetes participants than in healthy subjects (p=0.047).

|  |  | **Diabetes Group (n=20) (mean±SD)** | **Control Group (n=33) (mean±SD)** | **p-value** |
| --- | --- | --- | --- | --- |
| **Heel** | VPT 200-Hz (ln) | 0.50 ± 1.49 | 0.64 ± 1.58 | .770 |
|  | VPT 30-Hz (ln) | 3.22 ± 0.90 | 2.75 ± 0.99 | .089 |
|  | Stiffness (N/mm) | 5.9 ± 1.1 | 5.5 ± 1.2 | .269 |
|  | Hardness (Sore Scale) | 33.4 ± 8.3 | 28.6 ± 7.9 | .042* |
|  | Thickness (mm) | 0.94 ± 0.25 | 0.78 ± 0.17 | .02* |
|  | Peak pressure (kPa) | 278.7 ± 65.1 | 625.4 ± 254.8 | <.001* |
|  | Pressure-time integral [(kPa)*s] | 83.0 ± 22.8 | 95.8 ± 31.5 | .120 |
| **MTH** | VPT 200-Hz (ln) | 0.34 ± 1.62 | 0.59 ± 1.78 | .613 |
|  | VPT 30-Hz (ln) | 2.58 ± 0.97 | 2.32 ± 1.03 | .351 |
|  | Stiffness (N/mm) | 7.7 ± 1.8 | 7.0 ± 2.8 | .348 |
|  | Hardness (Sore Scale) | 27.1 ± 7.5 | 29.1 ± 14.0 | .493 |
|  | Thickness (mm) | 0.78 ± 0.16 | 0.72 ± 0.10 | .351 |
|  | Peak pressure (kPa) | 311.2 ± 115.1 | 232.4 ± 147.8 | .047* |
|  | Pressure-time integral [(kPa)*s] | 122.9 ± 55.5 | 115.3 ± 63.9 | .660 |

Abbreviations: MTH - metatarsal head; VPT – vibration perception threshold; SD - Standard Deviation *significant difference
